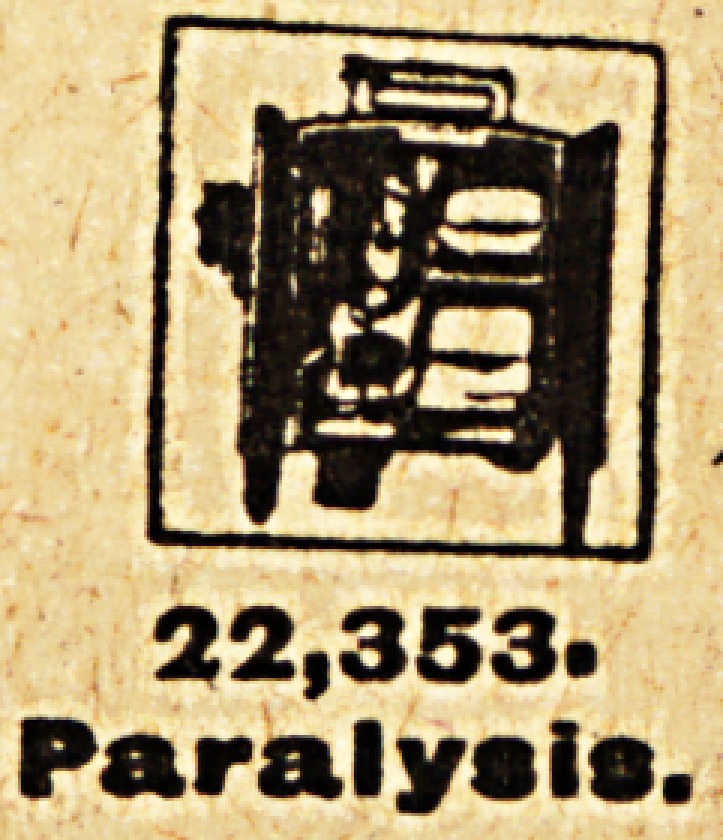# Over 1,600,000 Sufferers Helped by Our Hospitals

**Published:** 1919-06-21

**Authors:** 


### Over 1,600,000 Sufferers Helped by our Hospitals.

**A SINGLE YEAR'S ROLL-CALL OF THE SICK.**

IN the last year for which complete figures are available, the immense total of *one million six hundred and twenty-seven thousand, nine hundred and twelve* patients were treated at the voluntary hospitals and dispensaries of London and the infectious hospitals of the Metropolitan Asylums Board. These figures include only the in-patient cases treated to a termination in the wards of the hospitals and the number of new out-patient cases treated in the out-patient departments and dispensaries, and may be taken as showing as nearly as possible the number of separate cases dealt with in the hospitals and dispensaries of the Metropolis. The total is approximately one hundred thousand less than in the previous year, and this figure is entirely in respect of the numbers of out-patients. The result is probably chiefly caused by the absence from home of a large number of working men, who would, in an emergency, apply for treatment at a voluntary hospital.

626,955. Surgical Patients.

**Patients Suffering from Surgical Diseases.**—Of the whole number of patients received by the hospitals and dispensaries, *six hundred and twenty-six thousand nine hundred and fifty-five* required surgical treatment in addition to those treated in the special departments and in the hospitals for diseases of the eye, nose, throat, ear, etc. "Surgical" diseases include not only all accidents such as broken or fractured bones, cuts, burns, and all manner of displacements, crushings, and injuries of sensitive parts and organs, but also abscesses, ulcerations, cancers, and tumours of all kinds. Cases requiring Finsen light and Röntgen ray and similar treatment are classed under this heading, and the provision and upkeep of efficient curative apparatus make a steady call upon the resources of a hospital whose work includes such treatment. Nearly one million patients are treated annually in the London hospitals for diseases requiring surgical treatment.

529,298. Medical Patients.

**Patients Suffering from Medical Diseases.**—*Five hundred and twenty-nine thousand two hundred and ninety-eight* persons received medical treatment. By medical diseases are meant those diseases which are situated either as to their source and origin or in their entirety in one or other of the three great cavities of the body. They include rheumatic fever, pneumonia, pleurisy, bronchitis, diseases of the stomach, bowels, liver, kidney, bladder, and pancreas, every kind of heart disease, many forms of brain injury, dyspepsia, constipation, the manifold diseases of the nervous system, and other ailments, many of them serious or dangerous to life, or at least to the useful existence of the individual. Unlike some surgical ailments with outward signs or symptoms, medical diseases are often out of sight; the diagnosis of their nature and extent, and the successful treatment of them, is dependent on the doctor's scientific knowledge. This knowledge is in the hospitals of London freely given to nearly seven hundred thousand patients by the foremost physicians of the day.

153,882. Eye.

**Patients Suffering from Eye Affections.**—*One hundred and fifty-three thousand, eight hundred and eighty-two* persons were treated in the special departments of the general hospitals or by the ophthalmic hospitals of London. The blessing of unimpaired vision is appreciated by all, and the economic value of preserving and improving sight is incalculable. By this specialised treatment many patients have been saved from becoming greatly handicapped in the business of life.

### THE ROLL-CALL OF THE SICK.—*continued.*

170.284. Children.

**Patients Treated at Special Hospitals for Children.**—The total of patients mentioned at the commencement of this article include *one hundred and seventy thousand two hundred and eighty-four* children sent from their own homes, where they could not be properly attended to, for treatment in the special hospitals for children. Of course, a great many more of our little ones were also treated at the general and other institutions. For obvious reasons the health of the rising generation is at the present time especially a valuable asset to the nation.

93,350. Women.

**Diseases of Women and Motherhood.**—*Ninety-three thousand three hundred and fifty* women were treated at the Metropolitan voluntary hospitals for those diseases which are peculiar to their sex, or in the lying-in hospitals, where the best of our midwives are employed and trained. The very heart and strength of the nation lies in the home life, and the soul of the home life is the woman—the mother. Never was there a time when healthy and happy mothers were more valuable to the country than at the present day when motherhood, always sacred, is unusually precious.

75,755. Ear and Throat.

**Patients Suffering from Diseases of the Ear, Nose, and Throat.**—At the special hospitals or special departments devoted to these diseases *seventy-five thousand seven hundred and fifty-five* patients were treated. The organs mentioned are intimately connected, and diseases and ailments affecting them involve temporary and often permanent impairment of the functions of hearing, swallowing, and breathing. Those in full health perhaps may find it difficult to understand, until experience has brought the fact home to them, what discomfort and loss of efficiency to the individual are caused by any affection of the ear, nose, or throat.

56,878. Skin.

**Patients Suffering from Diseases of the Skin.**—During the year *fifty-six thousand eight hundred and seventy-eight* persons were treated for skin diseases in London. Although in these cases there is not, as a rule, the pain, or the danger to life, nor even such risk of permanent disablement as is the case with many other diseases, our sympathy is largely called for. The discomfort caused to the sufferer from these ailments and to his immediate friends is often considerable, and the expense of adequate treatment is so great that it is difficult to realise what the result would be were there no hospitals for the relief of this kind of patient.

41,350. Consumption.

**Patients Suffering from Consumption.**—*Forty-one thousand three hundred and fifty* patients suffering from phthisis or consumption, or diseases of the chest, were treated in the hospitals of London during the year. Most of us have seen something of the ravages and cruelty of consumption, and all dread this terrible disease, which may be called the curse of our climate. By it neither persons nor estate, rich nor poor, old nor young, are respected.

28,091. Fever.

**Patients Suffering from Fever.**—The number of patients treated was *twenty-eight thousand and ninety-one.* The diseases here classified include scarlet fever, diphtheria and measles; the latter has prevailed to such an extent in London during recent years that more deaths have occurred from it than from scarlet fever. Many families have experienced the emergency when the value of the Fever Hospital is appreciated.

22,353. Paralysis.

**Patients Suffering from Paralysis, Epilepsy, and Nervous Diseases.**—*Twenty-two thousand three hundred and fifty-three* persons stricken by paralysis, epilepsy, neuritis, neuralgia, neurasthenia and kindred ailments received treatment at the general hospitals and hospitals devoted to these maladies. The hurry and stress of modern life reap a terrible harvest, especially in a vast centre like London, where it is impossible to dissociate nervous breakdown from the toil and hurry of existence. No disease is more sudden than paralysis, surely none claiming more pity for its victims, often struck down without the slightest warning.

This is the story and roll-call of an army of sufferers, not far short of millions, who have received treatment over an extended period within the year under review. Again they claim our sympathy and help. To the vigorous, to those in health who are able to provide for their dependants, to those who know what ill-health means, who have suffered from disease of one kind or another, and who, either in the hospital or under the skill and care of the doctors and nurses trained in the hospitals, have been restored to health and usefulness, we confidently appeal on behalf of the London hospitals.

### THE ROLL-CALL OF THE SICK.

| | | | |
|---|---|---|---|
| Sufferers needing Surgical Aid | 626,955 | Sufferers from Skin Diseases | 56,878 |
| Sufferers needing Medical Care | 52[illegible],298 | Consumptives | 41,350 |
| Sufferers from Eye Troubles | 153,882 | Fever Patients | 28,091 |
| Diseases of Women | 93,350 | Paralysis and Epilepsy | 22,353 |
| Diseases of the Ear, Nose, and Throat | 75,755 | Total | 1,627,912 |

## Figures and Tables

**Figure f1:**
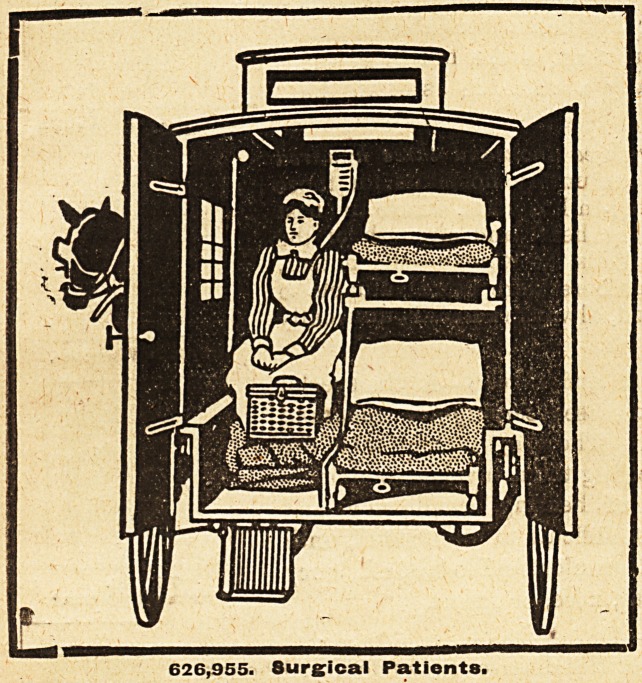


**Figure f2:**
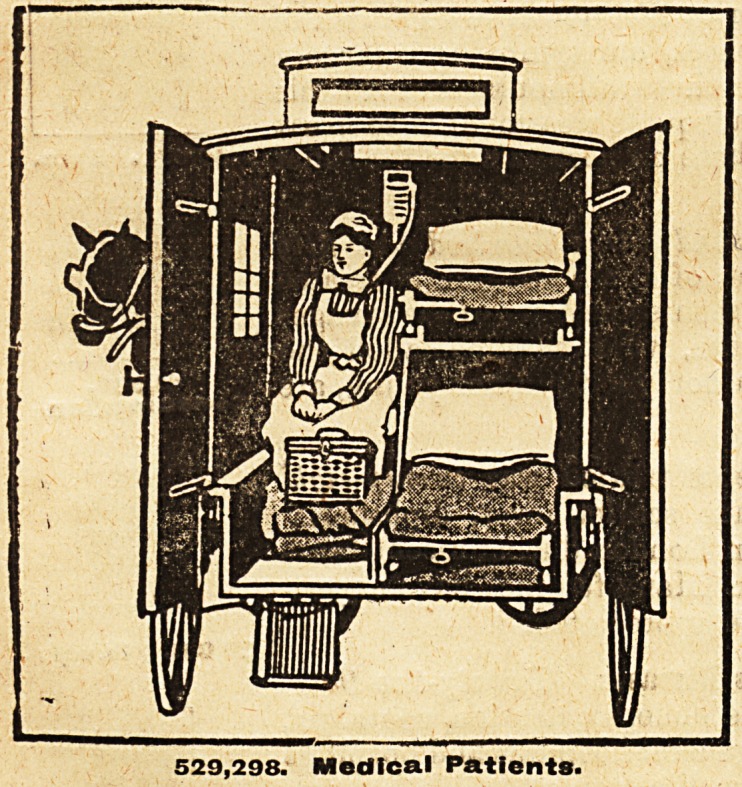


**Figure f3:**
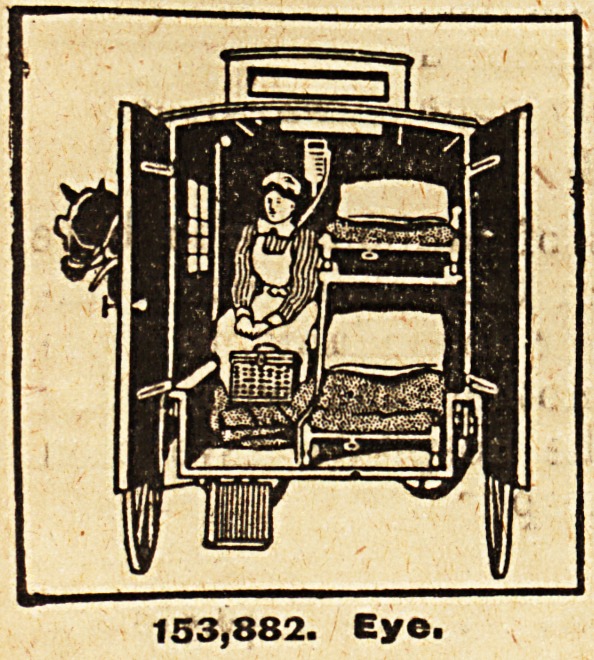


**Figure f4:**
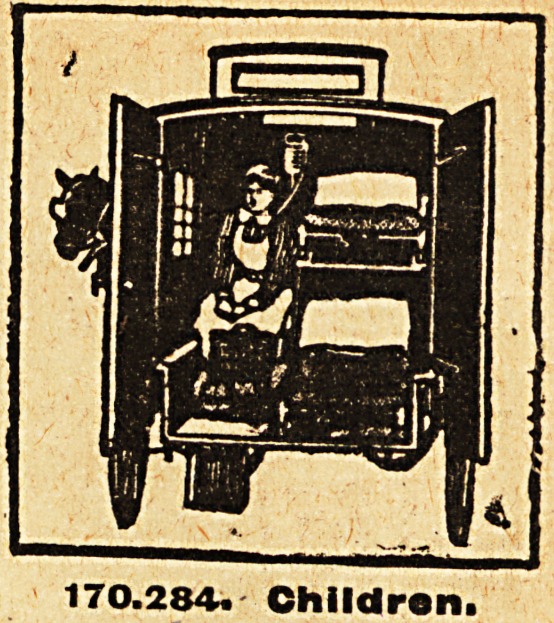


**Figure f5:**
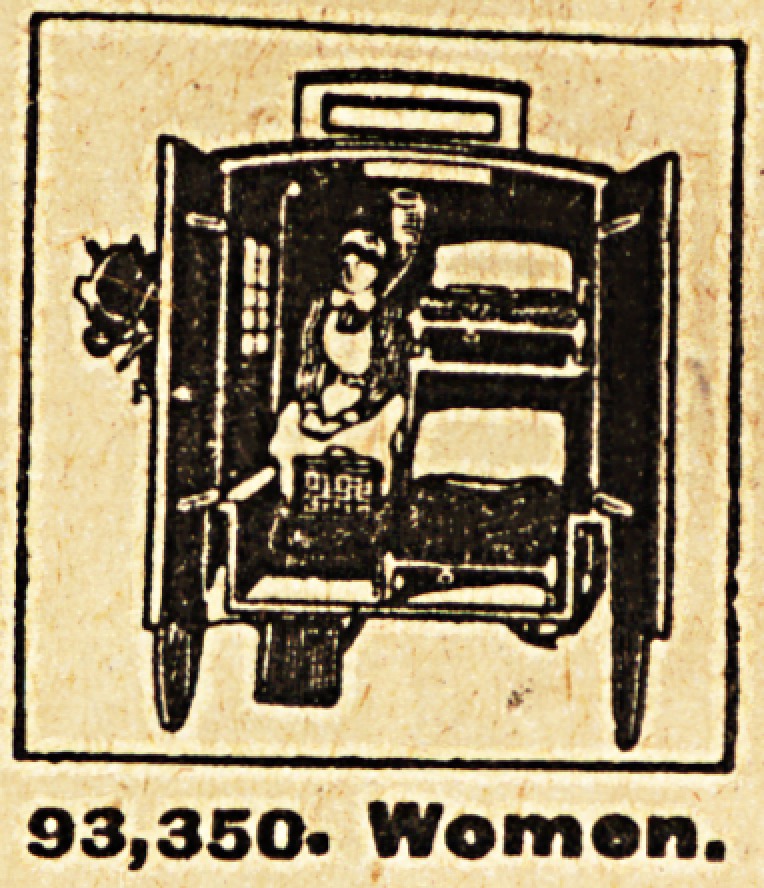


**Figure f6:**
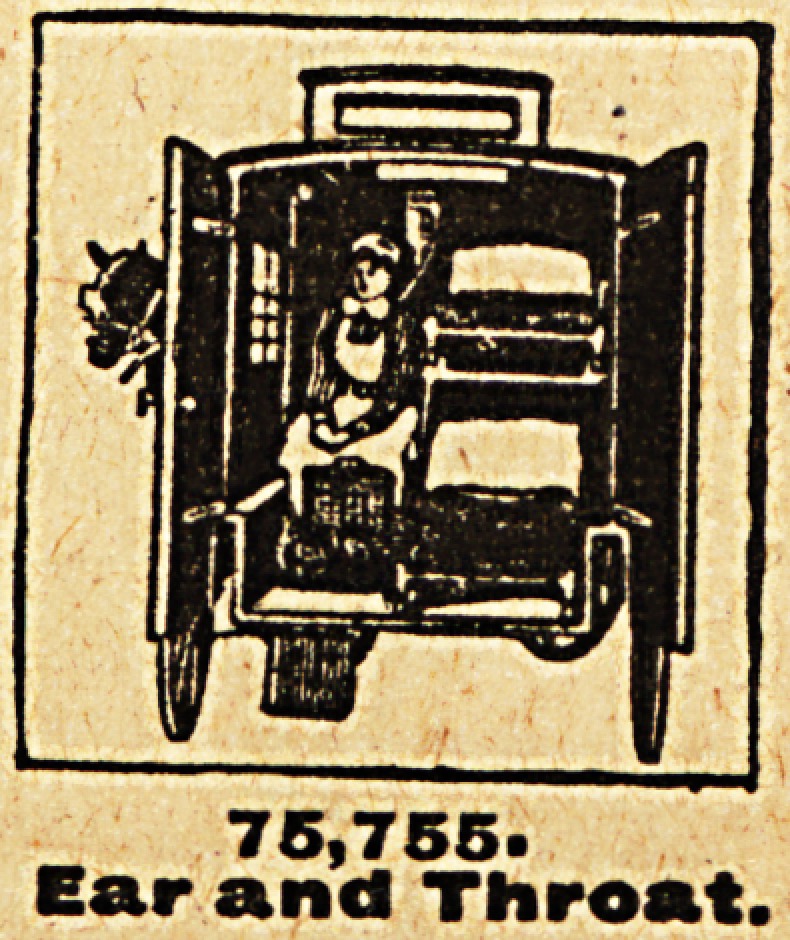


**Figure f7:**
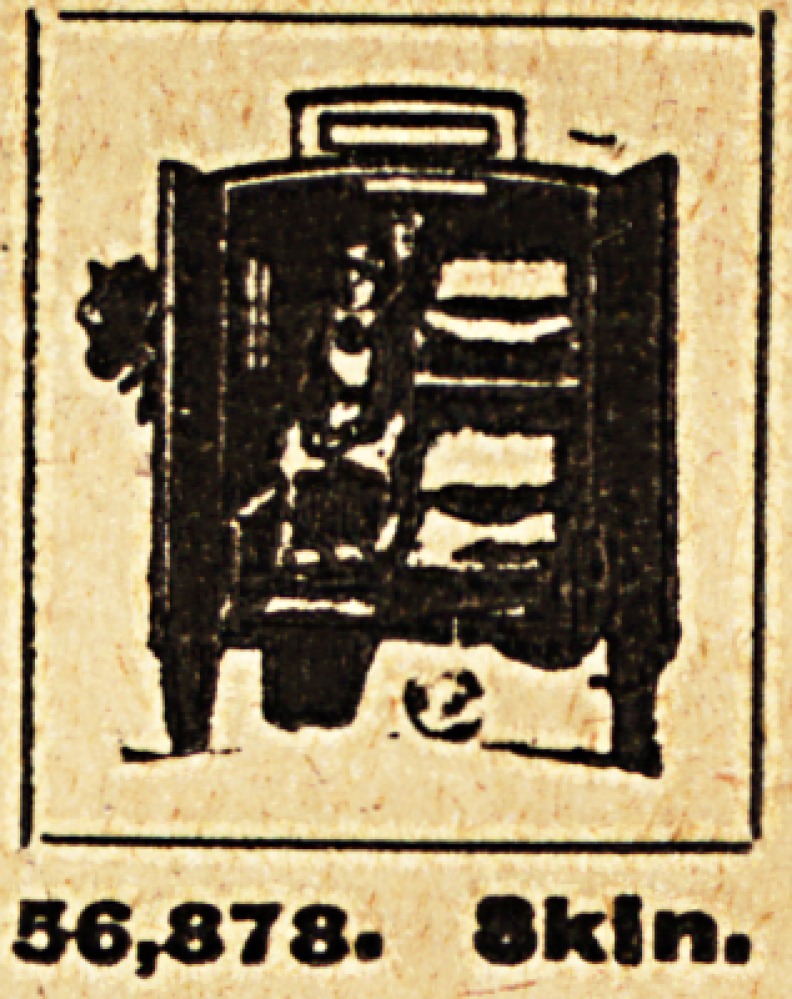


**Figure f8:**
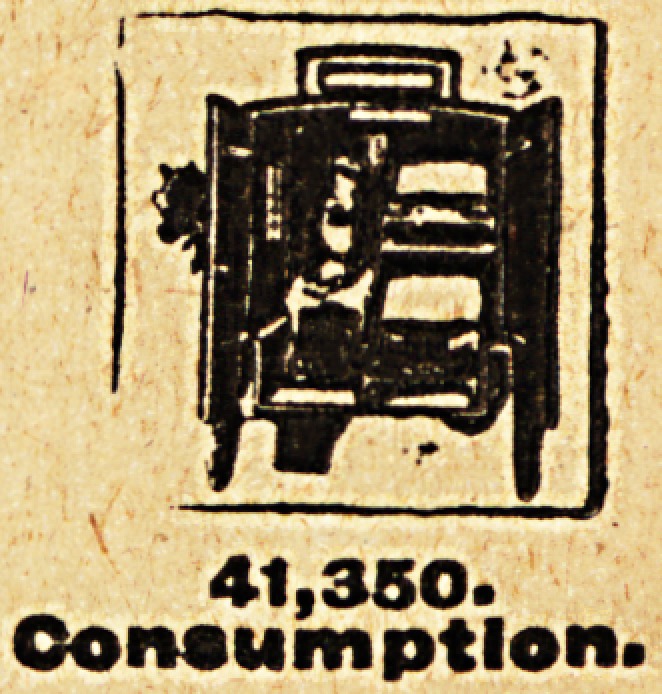


**Figure f9:**
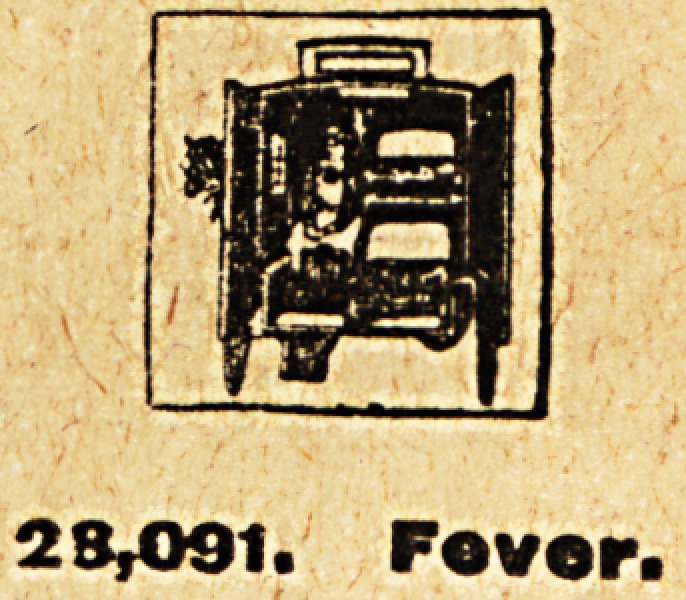


**Figure f10:**